# Washington's Tooth

**Published:** 1890-10

**Authors:** 


WASHINGTON'S TOOTH.—Isaac J. Greenwood, of New York, is exhibiting a tooth in a glass case. The tooth is mounted in gold. Above it hangs this extract from Mr. Greenwood's father's will: "I give and bequeath to my eldest son, Isaac John Greenwood, forever, all the curios, medallions, snuff-boxes, Gen. Washington's tooth and the under false jaw of teeth made for him by my late father, John Greenwood." The tooth, a large-sized molar, yellow from use and age, was the last one removed from the illustrious Washington's under jaw, according to the diary of Mr. Greenwood's father. In another glass case Mr. Greenwood exhibits a letter from Gen. Washington recording the remittance of $16 for a "false jaw." The letter was dated from Mount Vernon, January 6, 1799.

